# Mitochondrial Genome of *Abramis brama orientalis* Reveals Dominant Role of Natural Selection over Mutation Pressure in Shaping Codon Usage Bias in Leuciscinae Fishes

**DOI:** 10.3390/ani16071102

**Published:** 2026-04-03

**Authors:** Cui-Lan Hao, Yuan-Yuan Yang, Nian-Wen Wei, Jia-Qi Zhao, Cheng Yue, He Sun, Adili Abudu, Jian-Yong Hu, Yue Zhuo

**Affiliations:** 1 College of Veterinary Medicine, Xinjiang Agricultural University, Urumqi 830052, China; yangyuanyuan2024@126.com (Y.-Y.Y.); weinw21615@126.com (N.-W.W.); yuechengxnd@aliyun.com (C.Y.);; 2 Xinjiang Regional Key Laboratory of Clinical Veterinary Medicine Research, Urumqi 830052, China; 3 Xinjiang Key Laboratory of New Drug Research and Development for Herbivorous, Urumqi 830052, China; 4 Xinjiang Uygur Autonomous Region Fisheries Development Center, Xinjiang Uygur Autonomous Region Fisheries Science Research Institute, Urumqi 831399, China; xjadili@163.com (A.A.);

**Keywords:** *Abramis brama orientalis*, mitochondrial genome, comparative genomics, phylogeny, codon usage bias

## Abstract

Codon usage bias (CUB) is a universal feature in both mitochondrial and ribosomal genes, shaped by the combined forces of mutation and selection, and serves as a valuable indicator of evolutionary processes and phylogenetic signals. However, comprehensive analysis of CUB is lacking in the Leuciscinae family. We sequenced and annotated the complete mitochondrial genome of *Abramis brama orientalis* and examined codon usage patterns in all Leuciscinae species, subsequently finding the dominant evolutionary forces and phylogenetic relationships. We performed a PR2 bias plot, neutrality plot, effective number of codons (ENC) vs. GC3, relative synonymous codon use (RSCU) clustering and Ka/Ks calculations for 22 Leuciscinae species. We constructed the phylogenetic trees of Leuciscinae using Bayesian inference and maximum likelihood on concatenated mitochondrial sequences. The complete mitochondrial genome of *Abramis brama orientalis* was 16,607 bp, with typical vertebrate structure and high A + T bias. The codons of protein-coding genes in Leuciscinae have a preference for ending in A/C. All protein-coding genes were under strong purifying selection (Ka/Ks < 1). RSCU patterns and phylogenetic analyses showed two lines of Leuciscinae in the RSCU, with *A. brama orientalis* being a monophyletic group with *A. brama*. The results demonstrate the strong role for selection in shaping mitochondrial codon usage in Leuciscinae, despite mutational biases. The study clarified the taxonomic status of *A. brama orientalis* and provided a framework for understanding molecular evolution in this ecologically important freshwater fish family.

## Simple Summary

To better understand the evolution and aid in the conservation of freshwater fish, we studied the mitochondrial genomes of the Leuciscinae group. We sequenced the complete mitochondrial genome of the *Abramis brama orientalis* and compared it with 21 related species. Our study aimed to investigate the relative influence of mutation pressure and natural selection on mitochondrial codon usage bias in Leuciscinae. The results clearly show that natural selection was the dominant evolutionary force in Leuciscinae, overriding the effects of random mutation. This study also clarified the precise evolutionary position of the *Abramis brama orientalis* and highlighted the primary role of natural selection in shaping the genetic makeup of these fish, providing a clearer framework for their study and conservation.

## 1. Introduction

Mitochondria are the energy source of most eukaryotic cells, possessing small circular DNA that is used in molecular evolution, phylogenetics, and population genetics. In vertebrates, the mitochondrial genome of fish have relatively conserved gene content and order, maternal inheritance, small effective population size, and relatively high evolution rate; these make it the standard molecular marker for species identification, phylogeography and higher-level phylogenetic reconstruction [1]. Codons are the core elements of protein translation in gene-coding regions, the codon usage bias (CUB) refers to the non-uniform use of synonymous codons encoded by the same amino acid during translation; this is direct result of the long-term interaction between mutation pressure, natural selection and genetic drift and plays a significant role in fine tuning translation efficiency and accuracy [2].

Teleost fishes have been studied for mitochondrial CUB and have shown strong AT bias in their mitochondrial genomes due to strand-asymmetric mutation pressure during replication and transcription [3]. This leads to a general preference for NNU and NNA synonymous codons and indicates a consistent bias towards A/T ending codons, reflecting the high AT content of mitochondrial DNA [2,3]. In the context of the family Leuciscinae, preliminary mitogenomic studies have identified distinctive codon usage patterns that deviate from the canonical A/T bias observed in many teleosts, suggesting lineage-specific evolutionary pressures that may be linked to unique ecological adaptations or metabolic demands [4]. Studies across various taxa, including Cyprinidae [4,5,6,7], Salmonidae [8], and Cichlidae [9], have confirmed this pattern, suggesting that it may be a common feature in fish mitochondrial evolution. Moreover, analyses based on the effective number of codons (ENC) and neutrality plot attempted to quantify the relative contribution of mutation pressure and natural selection. In early work, Sharp et al. have indicated that mutation pressure may have played the dominant role in mitochondrial CUB [10]. However, an increasing number of studies suggest that natural selection (particularly selection for translation efficiency and accuracy) plays a significant and often predominant role in many species [11,12]. For instance, distinct CUB patterns have been linked to adaptation to extreme environments in some deep sea fish [13], indicating the role of natural selection. Phylogenetic signals in CUB have been observed in several fish families, indicating that codon usage patterns might reflect evolutionary relationships [14,15,16,17,18].

Over the past decade, several fish taxa have been characterized by the use of metrics such as RSCU, ENC, and correspondence analysis to quantify bias and speculate about its root causes [19,20]. Analyses of economically important species such as the *Epinephelus fuscoguttatus* have demonstrated a relatively weak CUB, with a subtle preference for A/C-ending codons, and have linked these patterns to selective pressures imposed by aquaculture practices [21]. Similarly, studies on *Ophisternon infernale* have highlighted distinct codon preferences in the mitochondrial genome that may reflect adaptations to hypoxic environments [22]. The development of robust bioinformatic pipelines, such as MitoFish/MitoAnnotator and CGView, have facilitated the accurate annotation and comparative analysis of thousands of circular mitochondrial genomes, enabling high-resolution assessments of codon bias across phylogenetically diverse groups [23,24]. In cichlid fishes, codon usage bias has been employed as an auxiliary marker for resolving deep phylogenetic relationships, revealing that certain codons (e.g., Leu1) exhibit exceptionally high usage bias, potentially reflecting adaptive translational strategies [25]. Recent whole-genome surveys of Stomatopoda species have underscored the conservation of codon bias patterns within specific lineages, while also identifying lineage-specific deviations that may be driven by unique selective regimes [26]. Collectively, these investigations have established a consensus that fish mitogenomes are subject to both mutational constraints (e.g., genome-wide AT richness) and selection for translational efficiency, with the relative contribution of each factor varying across taxa [27].

However, the CUB of mitochondrial are not clear in fish, most studies focus on general taxonomy comparisons or model species, and neither family- nor species-specific analysis is done at the family or genus level. For example, while previous investigations have characterized the mitogenomic features of individual Leuciscinae species, and comparative analyses have revealed conserved genome organization and synonymous codon usage across the family Leuciscinae [28], several critical questions remain unanswered, the relationship between codon usage patterns and phylogenetic divergence is not clear within Leuciscinae, and the relative contributions of mutation pressure versus natural selection to CUB have not been quantified across different Leuciscinae clades. Although the dichotomy of mutation pressure and natural selection is known, the relative contribution of mutation pressure and natural selection is often unclear and seems to differ by the lineage-specific adaptive evolution and historical phylogenetic divergence. The integrating CUB analysis with assessments of selective pressure (e.g., Ka/Ks ratios) and the phylogenetic inference to build a unified evolutionary framework are areas requiring further exploration.

The family Leuciscinae is a large and ecologically important group of freshwater fishes. The *Abramis brama orientalis* (Berg, 1949) (Leuciscinae, *Abramis*) was originally distributed in the Caspian Sea, White Sea and other waters in Europe [29,30] and is a kind of Leuciscinae fish with a flat body, short head and narrow and bulging back. The complete mitochondrial genome of *A. brama orientalis* (GenBank accession no.: KC894466) has previously been reported [31]. We sequenced, assembled, and annotated the complete mitochondrial genome of *A. brama orientalis* again in this study, and focused on the family Leuciscinae. This study provided a systematic, integrated analysis of mitochondrial genome structure, codon usage bias, evolutionary constraints, and phylogeny in the Leuciscinae. By clarifying the dominant evolutionary forces shaping their mitochondrial CUB and elucidating the phylogenetic utility of these patterns, this study aimed to provide new insights into the molecular evolution of this ecologically significant fish group and contribute to a broader understanding of adaptive evolution in vertebrate mitochondrial genomes.

## 2. Materials and Methods

### 2.1. Samples and DNA Extraction

A single individual of *A. brama orientalis* was collected from a site on the Irtysh River in Xinjiang, China (47°96′ N, 85°93′ E). After collection, the muscle tissue of *A. brama orientalis* was immediately fixed in 95% ethanol and subsequently stored at a temperature of −20 °C until nucleic acid extraction. Genomic DNA was isolated from the tissue at Nanjing Personal Gene Technology Co., Ltd. (Nanjing, China). The extracted DNA was electrophoresed on a 1% agarose gel to check for purity and integrity.

### 2.2. Genome Sequence Assembly and Annotation

The mitogenome *A. brama orientalis* was sequenced by the Illumina NovaSeq platform (Illumina, Inc., San Diego, CA, USA) high throughput sequencing system in Nanjing Personal Gene Technology Co., Ltd. (Nanjing, China). With the traditional whole-genome shotgun sequencing strategy [32], we used 1 μg of normalized DNA to prepare a paired-end short-insert library (400 bp). Initial raw data quality was assessed using FastQC software (https://www.bioinformatics.babraham.ac.uk/projects/fastqc/, accessed on 1 December 2025). Preprocessing of reads involved the removal of adapter sequences with AdapterRemoval (v2) [33] and subsequent quality filtering by SOAPec (v2.01) [34], which employs a Kmer-frequency-based algorithm. De novo assembly of the cleaned reads was performed independently by SPAdes (v3.9) and A5-miseq (v20150522) to generate primary contigs and scaffolds [35,36]. Putative mitochondrial genome sequences were identified from the filtered read pool via a BLAST (https://blast.ncbi.nlm.nih.gov, accessed on 1 December 2025) search. The assembled mitochondrial sequence was then subjected to base correction using Pilon software (v1.18) [37]. For annotation, the MITOS WebServer was utilized in the vertebrate mitochondrial genetic code (02) and default parameters [38]. The secondary structures of 22 tRNAs in *A. brama orientalis* were predicted by the MITOS WebServer [38]. The mitochondrial genome map of *A. brama orientalis* was initially generated using the web server OGDRAW (https://chlorobox.mpimp-golm.mpg.de/OGDraw.html, accessed on 20 March 2026) [39].

### 2.3. Data Retrieval and Analysis

The mitochondrial genome sequences of 22 species within the family Leuciscinae were retrieved from the NCBI database (https://www.ncbi.nlm.nih.gov/, accessed on 20 March 2026); corresponding species details are provided in Table 1.

For every mitochondrial genome sequence, nucleotide composition, amino acid usage, AT skew, GC skew, and relative synonymous codon usage (RSCU) were conducted by utilizing PhyloSuite v2 [40].

The RSCU values quantify codon usage bias, where a value below 1 signifies less frequent usage than expected, and a value above 1 indicates more frequent usage. In this analysis, codons with an RSCU exceeding 1.6 were classified as overrepresented, while those below 0.6 were considered underrepresented [41]. A clustering heatmap based on RSCU values was constructed using Origin 2021, the clustering method is selected as “Group Average”, and the distance measurement is chosen as “Euclidean Square” to measure the similarity among different codon usage patterns.

Additional indices of codon usage and synonymous codon usage bias were calculated with CodonW 1.4.2 [42], including overall GC content, the GC composition at the 1st and 2nd codon positions (GC12), the GC content specifically at the synonymous 3rd position (GC3), and the individual nucleotide frequencies (A, T, C, G) at this 3rd codon position, denoted as A3, T3, C3, and G3. The effective number of codons (ENC) was calculated, ranging theoretically from 20 to 61, [42]. A score of 20 signifies that only one codon is utilized for each amino acid, whereas a score of 61 indicates completely uniform usage across all synonymous codons [43]. Values below 35 are interpreted as signifying a pronounced codon preference, while scores exceeding 35 suggest a weaker bias. Furthermore, the presence of GC3s (GC content at synonymous third codon positions, excluding methionine, tryptophan, and termination codons) [43,44] was computed as an indicator of compositional bias.

**Table 1:** The species, GenBank accession numbers, and length of mitogenomes used in this study.

No.	Organism	Length [bp]	ID	Genus	Reference
1	*Abramis brama*	16,607	NC_020356.1	*Abramis*	[45]
2	*Abramis brama orientalis*	16,607	ON123737.1	*Abramis*	This study
3	*Abramis brama orientalis*	16,610	KC894466	*Abramis*	[31]
4	*Acanthobrama persidis*	16,613	NC_031562.1	*Acanthobrama*	--
5	*Alburnus alburnus*	16,605	NC_008659.1	*Alburnus*	--
6	*Alburnus mossulensis*	16,604	NC_031573.1	*Alburnus*	--
7	*Alburnus istanbulensis*	16,612	NC_019574.1	*Alburnus*	[46]
8	*Alburnus tarichi*	16,602	NC_019575.1	*Alburnus*	[47]
9	*Aspiolucius esocinus*	16,607	NC_031563.1	*Aspiolucius*	--
10	*Blicca bjoerkna*	16,605	NC_020355.1	*Blicca*	[45]
11	*Iberochondrostoma lemmingii*	16,601	NC_008108.1	*Iberochondrostoma*	[48]
12	*Leuciscus burdigalensis*	16,607	NC_029426.1	*Leuciscus*	[49]
13	*Leuciscus baicalensis*	16,606	NC_024528.1	*Leuciscus*	[50]
14	*Leucaspius delineatus*	16,602	NC_020357.1	*Leucaspius*	[45]
15	*Leuciscus oxyrrhis*	16,607	NC_029425.1	*Leuciscus*	[49]
16	*Leuciscus idus*	16,603	NC_063524.1	*Leuciscus*	--
17	*Leuciscus waleckii*	16,605	NC_018825.1	*Leuciscus*	[51]
18	*Notemigonus crysoleucas*	16,583	NC_008646.1	*Notemigonus*	[50]
19	*Pachychilon pictum*	16,602	NC_033920.1	*Pachychilon*	--
20	*Pelecus cultratus*	16,610	NC_008663.1	*Pelecus*	[50]
21	*Rutilus rutilus*	16,606	AP010775.1	*Rutilus*	[52]
22	*Scardinius erythrophthalmus*	16,607	NC_031561.1	*Scardinius*	--
23	*Vimba melanops*	16,604	NC_031539.1	*Vimba*	--
24	*Diptychus maculatus*	16,764	ON872378.1	*Diptychus*	[6] Outgroup
25	*Gymnodiptychus dybowskii*	16,677	NC_028544.1	*Gymnodiptychus*	[53] Outgroup

### 2.4. PR2 Plot Analysis

The potential influence of mutational pressure versus selection was conducted by the parity rule 2 (PR2) on codon usage. This method employs a plot where the ordinate represents AT bias (A/(A + T)), and the abscissa represents GC bias (G/(G + C)) [54]. The plot central point indicates an absence of codon usage bias, where A=T and G=C. For individual genes, plotting these calculated bias values provides a visual representation of the nucleotides at the 3rd codon position [55]. The resulting plot is informative, as the distribution and distance of points from the central origin reveal both the direction and the magnitude of bias present in each gene’s codon usage pattern.

### 2.5. Analysis of Evolutionary Neutrality

An evolutionary neutrality analysis was conducted, commonly visualized through a neutrality plot, and was generated using GraphPad Prism 8.0.2 (GraphPad Software, San Diego, CA, USA; https://www.graphpad.com, accessed on 20 March 2026). The GC content of 3rd synonymous codon position (GC3) was plotted on the *x* axis and the average GC content of the 1st and 2nd codon positions (GC12) were plotted on the *y* axis. Each data point corresponds to an individual gene or species.

The relationship between GC12 and GC3 was evaluated using linear regression in SPSS 18.0 [56]. The relationship between GC12 and GC3 was tested by Pearson correlation analysis and linear regression analysis, in order to determine whether mutation pressure or natural selection was driving codon usage preference. The linear correlation between GC12 and GC3 was tested using the Pearson correlation coefficient, the correlation coefficient (r) reflects the tightness of the linear correlation between the two groups of data, and the values range from −1 to 1, the *p* values indicate that the correlation coefficient (r) is significant when they are not 0, with a threshold of 0.05. A statistically significant correlation (*p* < 0.05) indicates that mutation pressure is the dominant force shaping codon usage bias. Conversely, a non-significant correlation suggests that natural selection plays a stronger role in regulating codon usage. The formula of linear regression analysis was a + b × GC3 = GC12, the model evaluation index was R^2^ (coefficient of determination) = SST/SSR = 1−SST/SSE, the b (slope) reflects the extent to which changes in GC3 affect changes in GC12, the R^2^ (coefficient of determination) reflects the regression model, which in turn explains the observed values, and ranges from 0 to 1. When GC12 and GC3 are significantly positively correlated (*p* < 0.05), and the slope b is close to 1 (b ≈ 1), this indicates that the change trend of base composition at the 1st, 2nd and 3rd positions in the genome is basically the same, in turn indicating that mutation (especially synonymous mutation) is the main driving force, and that the selection pressure is relatively weak. Thus, mutational pressure dominates. When GC12 and GC3 are significantly positively correlated (*p* < 0.05), but slope b is significantly less than 1 (b << 1), then, although the two are correlated, the change of position 12 (mainly constrained by selection) is much smaller than that of the 3rd position (large degree of mutation freedom), indicating that the 1st and 2nd positions are subject to strong selection constraints (such as translation efficiency, tRNA availability, etc.) and that natural selection is the main force, indicating that natural selection is dominant. When GC12 is not significantly correlated with GC3 (*p* > 0.05), the base composition of the 1st, 2nd and 3rd positions has changed independently, something which might be affected by different evolutionary pressures (such as complex balancing selection or species-specific genomic structure) and so indicating that there was no significant correlation [57,58,59].

### 2.6. Analysis of ENC–GC3s Plots

The ENC ratio is used to measure the relative deviation between the actual effective codon number (ENC) and the theoretical expected value (expected ENC) of a gene: ENC ratio = (ENCexp − ENCobs)/ENCexp [60]. The formula, ENC = 2 + GC3s + 29/[GC3s^2^ + (1 − GC3s^2^)], was used to calculate the expected value of ENC [43]. The ENC plot was generated by Nanjing Genepioneer Biotechnology Co Bioinformatics Cloud (http://112.86.217.82:9929/#/home, accessed on 20 March 2026). When the ENC ratio is closer to 0, the actual ENC is closer to the expected value, and the gene point is closer to the standard curve. The further away the ENC ratio is from 0, the more the gene point deviates from the standard curve, −0.05 ≤ ENC ratio ≤ 0.05, indicating that gene spots are close to the standard curve, and that mutations are the main driving force. ENC ratio < −0.05 or ENC ratio > 0.05, indicates that gene points deviate significantly from the standard curve and that selection is the main driving force [60]. A binomial distribution test was used to test whether the proportion of genes that deviated significantly from the standard curve (selection dominance) was significantly higher than expected by chance (50%). All statistical analyses were completed using SPSS 18.0 software [56].

### 2.7. Evolutionary Rate

The average rate of non-synonymous substitutions (Ka) and the average rate of synonymous substitutions (Ks) can be used to assess variations in PCGs in closely related species [61]. The Ka, Ks, and the average ratio of Ka/Ks were calculated by DnaSP V 5.0 [62] in each PCG. If the Ka/Ks < 1, this indicates purification or negative selection; if the value of Ka/Ks = 1, this indicates neutral; and if the Ka/Ks > 1, this indicates a positive selection; finally, when Ka/Ks is close to 1, this indicates low selection pressure [63].

### 2.8. Phylogenetic Analysis

The mitochondrial genomes of 22 species were selected in family Leuciscinae, with *Diptychus maculatus* and *Gymnodiptychus dybowskii* chosen for the outgroup. For all 22 species, nucleotide sequences of 37 mitochondrial genes (PCGs, rRNAs, and tRNAs) were extracted from their complete mitogenomes using PhyloSuite v2 [40]. Multiple sequence alignments of 13 PCGs were performed with MAFFT v7.313 [64]. Then, codon alignment of PCGs was performed using MACSE v2.03 [65], and the resulting alignments were pruned on Gblocks [66] under default parameters to retain conserved blocks. For both Bayesian inference (BI) and maximum likelihood (ML) phylogenetic reconstructions, optimal partitioning strategies and nucleotide substitution models were identified separately, with the BIC and AICc respectively applied for the model comparison performed in PhyloSuite v2 using ModelFinder (integrated within IQ-TREE v1.6.8) (Table S1) [67]. Bayesian analysis was executed with MrBayes v3.2.6 [68], with four MCMC chains operating independently for 2 million generations, sampling every 1000 generations. To ensure stationarity, the initial 25% of sampled generations were excluded as burn-in. The Bayesian analysis was run until stationarity was achieved, with the average standard deviation of split frequencies falling below 0.01, ESS values exceeding 200, and PSRF values approaching 1. Concurrently, ML trees were built using IQ-TREE v1.6.8 [69] with 5000 fast bootstrap replicates to assess nodal support. The final phylogenetic trees from both methods were subsequently visualized and displayed on iTOL web server (http://itol.embl.de, accessed on 21 March 2026).

## 3. Results

### 3.1. Characteristics of Mitochondrial Genome

**Figure 1:**
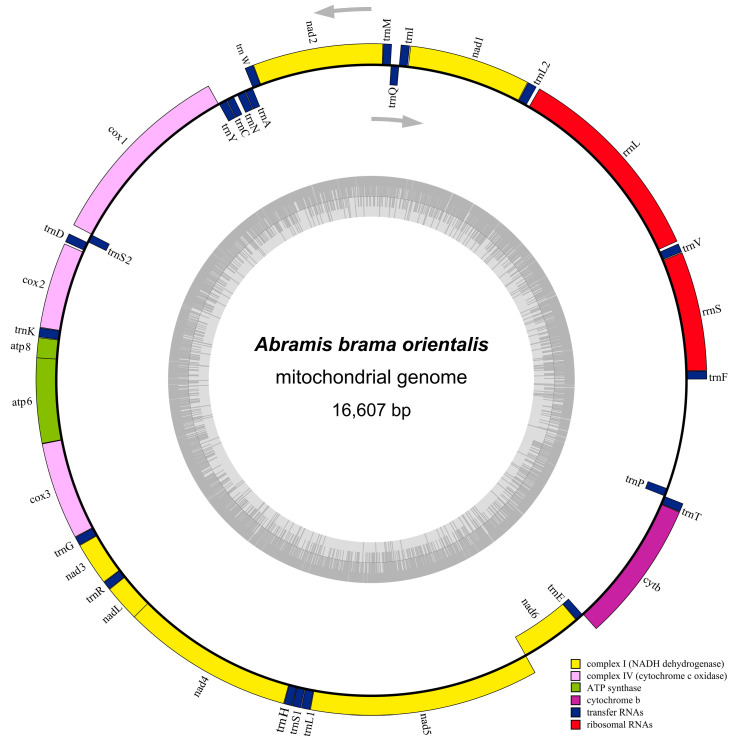
The circular map of the mitochondrial genome of *A. brama orientalis*. The inner ring is the light strand and the outer ring is the heavy strand. Protein-coding, tRNA and rRNA genes are shown with standard abbreviations. Arrows indicate the direction of transcription. The inner gray circle represents the GC content.

To confirm the mitochondrial genome organization of *A. brama orientalis*, we performed sequencing, assembly, and annotation using the Illumina NovaSeq high-throughput platform, consistent with the report by Qi [31]. As shown in Figure 1 and Table 2, the mitochondrial genomes of *A. brama orientalis* shared a highly conservative gene arrangement, it had a typical circular mitochondrial DNA with 16,607 bp in length (GenBank No: ON123737), including 13 PCGs, 22 tRNAs, 2 rRNAs, and 2 non-coding regions: origin of light-strand replication (OL) and control region (D-loop). The *nad6* protein-coding gene and eight tRNAs (*trnA*, *trnC*, *trnE*, *trnN*, *trnP*, *trnQ*, *trnS2*, and *trnY*) were encoded on the light strand. The remaining 28 genes were located on the heavy strand. In addition, there were seven overlaps in the mitochondrial genome of *A. brama orientalis* (ranging from 1 bp to 7 bp, Table 2), the overlap regions between *atp8* and *atp6*, *atp6* and *cox3*, *nad4L* and *nad4*, and *nad5* and *nad6* were 7, 1, 7, and 4 nucleotides, respectively. The PCGs of *A. brama orientalis* were 10,974 bp in length, and the *nad5* gene (1836 bp) was the longest in PCGs, with the *atp8* gene (165 bp) being the shortest. These results indicate that *A. brama orientalis* shares similarities in conserved structure with Cyprinidae species.

The start codons and stop codons, which indicate the end of protein synthesis in all organisms, play an important role in the transcription and translation process. To further investigate the features of initiation and termination codons in the mitochondrial genome of *A. brama orientalis*, we conducted a systematic analysis of their distribution patterns. As shown in Table 2, the start codon ATG was conservatively used in 12 PCGs, while GTG was used in *cox1*. Among the PCGs, one gene (*cytb*) used the incomplete termination codon T, four genes (*nad2*, *atp8*, *nad3*, and *nad4*) utilized the typical termination codon TAG, while eight genes (*nad1*, *cox1*, *cox2*, *atp6*, *cox3*, *nad4L*, *nad5*, and *nad6*) employed the typical termination codon TAA. These results reveal the unique characteristics of *A. brama orientalis* mitochondrial genes, with such traits potentially associated with the evolution of mitochondrial gene regulation, while reflecting its similarity to other Leuciscinae species.

### 3.2. Genome Structure and Composition of Mitogenomes in Leuciscinae Species

**Figure 2:**
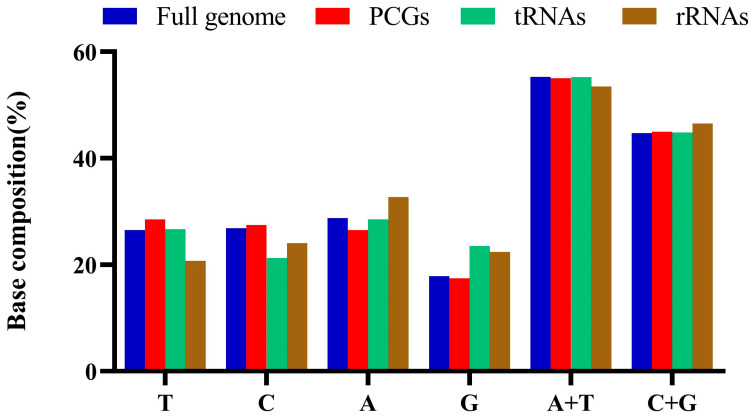
Average base composition of full genome, PCGs, tRNAs and rRNAs of mitogenomes in Leuciscinae.

To understand the genome structure and composition of mitogenomes in species of the family Leuciscinae, we studied the size and AT content of PCGs, tRNA and rRNA from 22 Leuciscinae species. The 22 Leuciscinae species had PCGs ranging from 11,412 to 11,496 bp, rRNAs from 2604 to 2650 bp, and tRNAs from 1531 to 1577 bp (Figure 2, Table S2). The A + T contents of PCGs, tRNAs and rRNAs were higher than 50% in Leuciscinae species. The contents of the four bases were A > C > T > G, T > C > A > G, A > T > G > C, A > C > G > T among the full genome, PCGs, tRNAs and rRNAs of Leuciscinae species, respectively. The A + T contents (53.5–57.3%) were higher than the C + G contents (42.8–46.5%) in the Leuciscinae species (Figure 2). These results indicate that the PCGs, tRNAs and rRNAs of Leuciscinae had AT base bias.

### 3.3. PR2 Plot and Neutrality Plot

**Figure 3:**
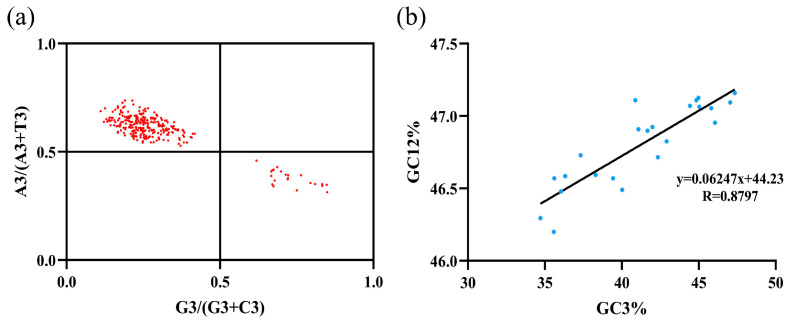
PR2 bias plot (**a**) and neutrality plot (**b**) of Leuciscinae. (**a**) PR2 bias plot showing the distribution of A3/(A3 + T3) versus G3/(G3 + C3) for 13 protein-coding genes across 22 Leuciscinae species in the four quadrants, the red dots represent PCGs of mitogenomes in Leuciscinae. (**b**) Neutrality plot examining the correlation between GC3 and GC12 across 22 Leuciscinae species, the blue dots represent species of Leuciscinae.

To evaluate the nucleotide composition bias and the relative influences of mutation pressure versus natural selection on codon usage in Leuciscinae, we performed PR2 bias and neutrality plot analyses based on 13 mitochondrial protein-coding genes. As shown in Figure 3a, the parity rule 2 (PR2) plot displays the relationship between A3/(A3 + T3) and G3/(G3 + C3) for each gene. The points were distributed across four quadrants, with the majority (56 out of 72 genes, 77.8%) located in the fourth quadrant, indicating a pronounced preference for T over A and for G over C at the third codon position. Only one gene fell into the second quadrant, while seven and eight genes were in the first quadrant and the third quadrant respectively, which indicated compositional biases towards T and G at the synonymous sites across the Leuciscinae mitogenomes.

To assess the relative contributions of mutation pressure and natural selection to codon usage bias. We measured the correlation between GC content at the 3rd codon position (GC3) and average GC content at the 1st and 2nd positions (GC12) by neutrality plot. As shown in Figure 3b, GC3 values ranged from 23.8% to 55.5%, while GC12 values ranged from 34.8% to 44.4%. A strong positive correlation was detected between GC3 and GC12 (Pearson’s r = 0.8797, *p* < 0.05), indicating that the 22 Leuciscinae species examined are subject to measurable mutation pressure. Linear regression analysis yielded a slope (b) of 0.06247 (regression equation: y = 0.06247x + 44.16), which was substantially lower than 1, the slope indicated that, for every 1% increase in GC3, GC12 increased by only approximately 0.062%. Consistent with this near-horizontal regression line, most data points deviated markedly from the diagonal (Figure 3b), where GC12 would be expected to covary proportionally with GC3 under uniform mutation pressure. These results indicate that, while a clear mutational bias exists—favoring T and G at the 3rd codon position—natural selection played the dominant role in shaping codon usage patterns in Leuciscinae mitochondrial genomes. The contribution of mutation pressure, although detectable, was comparatively limited.

### 3.4. ENC–GC3s Plot and Neutrality Plot

**Figure 4:**
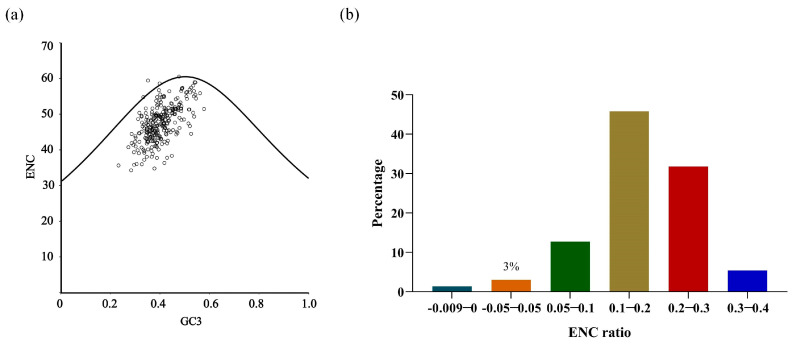
ENC–GC3s plot of 13 PCGs (**a**) and ENC ratio (**b**) in Leuciscinae. (**a**) The solid curve represents the expected ENC values under the assumption that codon usage bias is solely determined by GC3s composition. Each circle represents a single gene. Circles located below the curve indicate that factors other than mutation pressure (e.g., natural selection) contribute to codon usage bias. ENC < 35 indicates a strong codon preference, ENC > 35 indicates weak codon preference. (**b**) ENC ratios within −0.05 to 0.05 indicate codon usage patterns primarily driven by mutation pressure.

To investigate the effect of the effective number of codons (ENC) and GC content at the third codon position (GC3) of 13 protein-coding genes (PCGs), we analyzed the correlation between these two parameters. As shown in Figure 4a, most of the points corresponding to or below the expected ENC–GC3 curve, suggesting that codon preference differs widely among genes, and that most of the points fell above 35 ENC (Figure 4a), which indicates a generally weak codon bias across the mitogenomes of Leuciscinae fish.

The distribution of ENC ratios (ENCobs/ENCexp) was also evaluated to assess the role of mutation pressure and natural selection in shaping CUB in Leuciscinae mitogenomes. As shown in Figure 4b, the distribution of ENC ratios was examined across 297 mitochondrial genes (13 PCGs from 22 species, with nad6 absent in three species). Among these, ENC ratios of only nine genes (3.0%) ranged from −0.05 to 0.05, indicating that their observed ENC values closely approximated those expected under pure mutation pressure. In contrast, the ENC ratios of the remaining 288 genes (97.0%) ranged from −0.1 to −0.3 or 0.1 to 0.4, reflecting substantial deviations from mutational equilibrium. A binomial test confirmed that the proportion of genes ranging from −0.05 to 0.05 was 97.0%, significantly higher than the 50% expected under random distribution (*p* < 0.001). The results indicate that codon usage patterns were not solely determined by mutation pressure for the vast majority of mitochondrial PCGs in Leuciscinae.

### 3.5. Analysis of RSCU in 22 Leuciscinae Species

**Figure 5:**
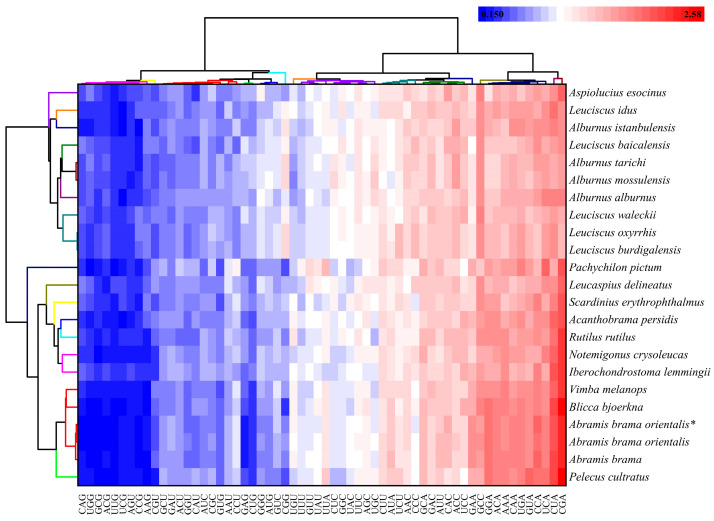
Hierarchical clustering and heat map of RSCU for Leuciscinae species, where color change from blue to red indicates increase in RSCU. Each square represents the RSCU value of a codon (shown in columns) corresponding to the species (shown in rows). The color coding varies from blue to red with low to high values of the RSCU respectively. *: Species sequenced in this study.

To understand the codon usage patterns among Leuciscinae species, we clustered species according to relative synonymous codon usage (RSCU) values of mitochondrial protein-coding genes. The heatmap is blue (low RSCU) to red (high RSCU) and the change in preference between species can be seen clearly within the studied taxa. As shown in Figure 5, Table S3, all species were classified into groups: *Pachychilon pictum* and *Aspiolucius esocinus* formed early diverging branches, followed by *Leucaspius delineatus* and *Pelecus cultratus*, and the remaining species diverged late. In groups, codon usage profiles were similar: synonymous codons ending in A or C represented RSCU > 1 (CGA, CUA, UCA, CCA, GUA) and favoring A-ending codons in most species. Codons ending in G (CAG, UCG, GCG, ACG, UUG,) had RSCU < 1, reflecting lower usage frequency.

Noticeably different behavior is observed between the two main clades. For example, codons GGC and UGC have higher RSCU (closer to or above 1) in Leuciscus than codons in Alburnus. Additionally, codon GGC has RSCU > 1 in some Alburnus and Leuciscus species but was avoided (RSCU < 1) in the other cluster. This pattern shows that, although Leuciscinae share a common A/C ending codon bias, lineage-specific codon preferences contribute to differences in RSCU profiles. Overall, the hierarchical clustering and heatmap analysis indicated that codon usage is phylogenetically structured, with distinct codon preference signatures distinguishing major species groups.

### 3.6. Selection Pressure

**Figure 6:**
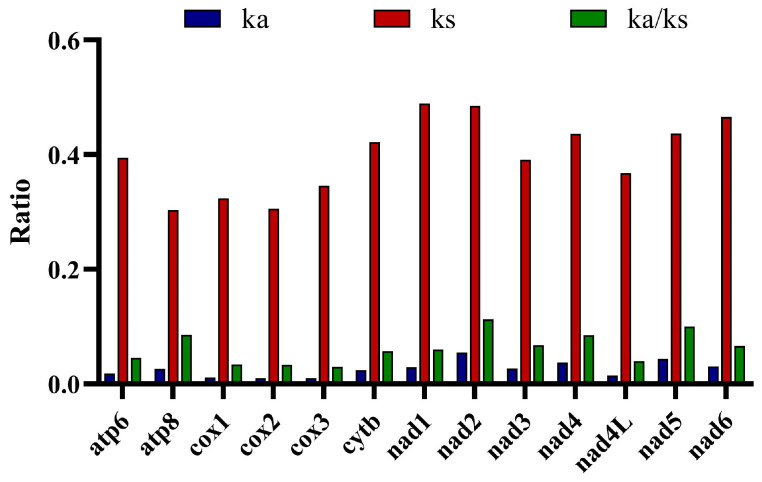
Comparisons of average Ka, Ks and Ka/Ks ratios for 13 PCGs in Leuciscinae.

In order to evaluate evolutionary constraints on protein-coding genes in the Leuciscinae mitogenomes, we calculated the average nonsynonymous substitution rate (Ka), synonymous substitution rate (Ks) and their ratio (Ka/Ks) of each of the PCGs (Figure 6). The Ka/Ks ratios of all genes were far below 1, ranging from 0.029 (*cox1*, *cox2*, *cox3*) to 0.112 (*nad2*), and thus indicating widespread purifying selection in the family.

Among the 13 genes, the Ka/Ks ratio value of *nad2* was highest (0.112), followed by *nad5* (0.100) and *atp6* (0.085). The Ka/Ks ratio values of *cox1* (0.034), *cox2* (0.033) and *cox3* (0.029) were lowest, reflecting stronger evolutionary conservation. The Mann–Whitney U test revealed significant differences between the cox gene (*cox1*–*3*) and the nad gene (*nad1*–*6*, *nad4L*)(*p* < 0.05), indicating that there are statistically significant differences in the selection pressure of these two gene groups. The Ka values of some genes were low (0.010–0.054), while those of *nad2* (0.054) and *nad5* (0.043) were highest. Ks values were consistently higher than Ka in all genes, ranging from 0.30 (*atp8*) to 0.49 (nad1), further indicating the predominance of purifying selection. The variation in Ka/Ks suggests differential selective pressures among genes. These results confirm that all 13 mitochondrial PCGs in Leuciscinae were under strong purifying selection, with gene-specific differences in evolutionary rate likely reflecting their distinct functional and structural roles in the oxidative phosphorylation pathway.

### 3.7. Phylogeny

**Figure 7:**
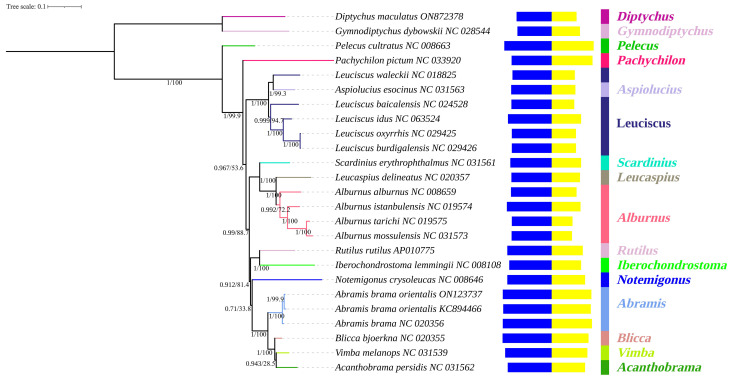
Phylogenetic tree constructed by BI and ML methods, based on the mitochondrial genome sequence of Leuciscidae fish. Numbers on branches represent posterior probabilities (BI) and bootstrap percentages (ML), respectively. The histograms are the GC skew (before) and AT content (after) of the mitochondrial genome, respectively. Taxonomic identity is shown to the right: Genus.

To further examine the relationship between *A. brama orientalis* and other Leuciscinae species, a phylogenetic tree was reconstructed to elucidate the relationships within the family Leuciscinae. As shown in Figure 7, the tree topology, supported by high posterior probability values (most nodes ≥ 0.99), reveals clear phylogenetic structuring among the examined species. The Leuciscinae species form a well-supported monophyletic clade, distinct from the outgroup *D. maculatus* and *G. dybowskii* (family Schizopygopsinae). Within the phylogeny of Leuciscinae, *Pelecus cultratus* occupied the basal position, indicating it is the earliest divergent species in this group.

The remaining Leuciscidae species diversified into two distinct clades: one comprising species of *Pachychilon*, *Aspiolucius* and *Leuciscus*, and the other consisting of *Scardinius*, *Leucaspius*, *Alburnus*, *Rutilus*, *Iberochondrostoma*, *Notemigonus*, *Abramis*, *Blicca*, *Vimba* and *Acanthobrama*. Notably, the genus *Leuciscus* is not monophyletic, with *L. waleckii* and *Aspiolucius esocinus* clustering closely. Similarly, species of *Alburnus* (*A. mossulensis*, *A. tarichi*, *A. istanbulensis* and *A. alburnus*) form a cohesive group with strong support, indicating their close phylogenetic affinity. The genera *Abramis* (*A. brama* and *A. brama orientalis*), *Blicca*, *Vimba* and *Acanthobrama* cluster together with high support, suggesting a recent convergence. *Rutilus rutilus* and *Iberochondrostoma lemmingii* are in the second major lineage but have relatively long branches, suggesting greater genetic divergence. The result indicates that genus *Leuciscus* may not be monophyletic in Leuciscidae and warrants further study.

## 4. Discussion

We present, for the first time, a detailed analysis of the mitogenomic structure, codon usage, evolutionary constraints and phylogenetic relationships of the Leuciscinae, including *A. brama orientalis*.

The mitochondrial genome of *A. brama orientalis* was the typical genome of teleost fishes (13 PCGs, 22 tRNAs, 2 rRNAs and a control region) with the typical sequence of proteins found in teleost fishes [1]. This high degree of structure preservation highlights the constraints of mitochondrial genome organization [70]. However, specific features were observed, such as the use of GTG as a start codon for *cox1* and incomplete termination codon (T) for *cytb*. The GTG start codon is less common than ATG but is often found in fish mitochondrial *cox1* and is thought to be a functionally equivalent post-transcriptional modification [71,72]. Incomplete stop codons are characteristic of vertebrate mitochondrial genomes the single T residue probably completes to a full TAA stop codon by polyadenylation of the mRNA transcript, a mechanism found in teleosts [73,74]. Intergenic overlaps (e.g., 7 bp between *atp8* and *atp6*) are associated with compact genome packaging and possibly co-transcriptional regulation [75].

The overall AT bias (A + T > 50%) follows the compositional bias of vertebrate mitochondrial DNA due to symmetric mutation pressures during replication and transcription [76] and could affect codon usage and amino acid composition [77].

The results of the CUB showed that natural selection was responsible for synonymous codon choice in Leuciscinae mitogenomes, although mutation pressure had a significant influence. The ENC–GC3 plot showed that most genes had weak to moderate bias (ENC > 35), which is consistent with other fish mitochondrial genomes where functional constraints limit strong codon choice [20]. The ENC ratio distribution indicated that the observed values differed significantly from expectations under pure mutational equilibrium strongly suggesting selective optimization [20,78]. This pattern is unlikely to arise from neutral processes, as mutational pressure would have pushed observed values closer to the expected curve. Instead, the deviation suggests that selection has acted to refine codon usage, possibly to balance efficiency and accuracy during translation [79]. What remains unclear is whether the strength of this selection varies across genes or environments, and whether certain functional categories are more constrained than others. Future work incorporating expression data could help clarify whether highly expressed genes are under stronger translational selection, as seen in some other vertebrate systems [80]. Comparative genomic analyses with closely related cyprinids may also reveal whether the observed optimization reflects lineage-specific adaptation or a broader constraint shared across the family.

The PR2 plot showed a strong bias towards T and G over A and C at the third codon position. This pattern is also reported in the mitogenomes of other cypriniforms [81,82] and may be due to mutational bias (strand-asymmetric replication) and selective preference for translational efficiency or accuracy [79,83]. Crucially, the neutrality plot showed a regression slope of only 0.062 between GC12 and GC3. This slope indicates that less than 7% variation in GC3 is due to uniform mutation pressure across codon positions, and that more than 93% are due to natural selection or other constraints [57,84]. This is consistent with other vertebrate mitochondria where selective constraints on protein structure and function dominate compositional evolution [17,85].

The hierarchical clustering based on RSCU values corresponded very well to the sequence relationships. Species in the *Alburnus*-*Leuciscus* cluster had a different preference for codons. This pattern hints that codon usage, while broadly conserved along evolutionary lines, may be fine-tuned by stabilizing selection—possibly to optimize translational speed or maintain co-adaptation with the nuclear-encoded tRNA pool [86,87]. It would also be worth investigating whether shifts in codon preference coincide with ecological transitions or habitat shifts, which could impose new demands on translational efficiency [88]. Addressing these questions would require denser taxon sampling and functional assays. The preference for A-ending codons between Leuciscinae is consistent with the overall AT richness of their mitogenomes and is common in AT-biased genomes [89]. Lineage-specific variations of the usage of some codons show that selective pressures may differ subtly between clades, possibly due to differential adaptation or genetic drift [90]. Although the codon usage analysis indicates selection, it does not identify specific selective agents (thermal adaptation, oxidative stress) or the role of tRNA abundance (which requires expression data) [91].

The Ka/Ks ratios for all 13 PCGs were lower than 1 and indicate that purifying selection is the dominant mode of evolution, eliminating deleterious mutations to maintain protein function [92], universal for core metabolic genes in animal mitochondria [93], though the degree of purifying selection differed significantly between genes. Respiratory complex genes (*cox1*–*cox3*) showed the lowest Ka/Ks values (0.029–0.034), suggesting they have the slowest evolutionary rate and are subject to the most stringent purifying selection. These genes encode critical subunits of the electron transport chain, where structural integrity is important [94]. Several NADH dehydrogenase subunits (*nad2* and *nad5*) were found to have a higher Ka/Ks ratio (0.100–0.112), suggesting they have a relatively fast evolutionary rate and may be under weak purifying selection or neutral evolutionary pressure.

The phylogenetic studies on complete mitogenomes support the monophyly of Leuciscinae and clarify the position of *A. brama orientalis*. Close clustering with *A. brama* (PP = 1) strongly supports its classification as a subspecies of *Abramis*, confirming previous morphological studies and limited molecular studies [95,96]. In addition, the tree also identified one instance of non-monophyly at the genus level (e.g., *Leuciscus*), the sister relationship between *L. waleckii* and *A. esocinus* recovered here revealed a paraphyletic *Leuciscus* with respect to *Aspiolucius*, echoing the work of Schönhuth et al. (2018) [97], which is based on the mitochondrial gene dataset (*cytb* + *cox1*), nuclear gene dataset (*Rag1* + *IRBP*), and a combined dataset (*Rag1* + *IRBP* + *cytb* + *cox1*). Perea (2010) [98] and Schönhuth (2018) [97] synonymized *Aspius* and *Aspiolucius* with *Leuciscus*, respectively. The monophyletic nature of the genus *Leuciscus* may still need further clarification. A frequent issue of cyprinid taxonomy is likely due to convergence in morphological convergence, rapid radiation and incomplete lineage sorting [99,100]. The clear separation of major lines in Leuciscinae provides a solid foundation for future systematic study. The correlation between phylogenetic relationships and nucleotide composition biases, particularly GC/AT skew patterns observed across specific branches, suggests that genome composition evolves in a lineage-specific manner and may be associated with diversification processes [101,102]. Although mitochondrial genomes provide information on maternal lineage and evolutionary rates, they offer a limited view. A full assessment of selection and phylogeny is essential to integrate nuclear genome data, thereby accounting for biparental inheritance and mito–nuclear coevolution [103].

## 5. Conclusions

This study presented the mitogenome for *A. brama orientalis* and a comparative analysis of codon usage across Leuciscinae. The mitogenome of *A. brama orientalis* was AT rich but retains the conserved vertebrate gene order; the codon usage bias was weak across the family Leuciscinae, and influenced by purifying selection, albeit to varying degrees that may reflect their functional importance. The mitogenome-based phylogeny corroborating *Abramis* relationships and revealing non-monophyly in some genera offer a basis for taxonomic revision.

## Figures and Tables

**Table 2:** Summary of the mitogenome of *A. brama orientalis*.

Feature	Position	Length (bp)	Intergenic Nucleotide	Initiation Codon	Stop Codon	Anticodon	Strand
*trnF*	1–69	69				GAA	H
*rrnS*	70–1026	957					H
*trnV*	1029–1100	72	2			TAC	H
*rrnL*	1120–2766	1647	19				H
*trnL2*	2792–2867	76	25			TAA	H
*nad1*	2869–3843	975	1	ATG	TAA		H
*trnI*	3848–3919	72	4			GAT	H
*trnQ*	3918–3988	71	−2			TTG	L
*trnM*	3990–4058	69	1			CAT	H
*nad2*	4059–5105	1047		ATG	TAG		H
*trnW*	5104–5174	71	1			TCA	H
*trnA*	5176–5244	69	−2			TGG	L
*trnN*	5246–5318	73	1			GTT	L
OL	5321–5351	31	2				H
*trnC*	5351–5417	67	−1			GCA	L
*trnY*	5419–5489	71	1			GTA	L
*cox1*	5491–7041	1551	1	GTG	TAA		H
*trnS2*	7042–7112	71				TGA	L
*trnD*	7116–7189	74	3			GTC	H
*cox2*	7203–7907	705	13	ATG	TAA		H
*trnK*	7894–7969	76	−14			TTT	H
*atp8*	7971–8135	165	1	ATG	TAG		H
*atp6*	8129–8812	684	−7	ATG	TAA		H
*cox3*	8812–9651	840	−1	ATG	TAA		H
*trnG*	9596–9667	72	−56			TCC	H
*nad3*	9668–10,018	351		ATG	TAG		H
*trnR*	10,017–10,085	69	−2			TCG	H
*nad4L*	10,086–10,382	297		ATG	TAA		H
*nad4*	10,376–11,758	1383	−7	ATG	TAG		H
*trnH*	11,758–11,826	69	−1			GTG	H
*trnS1*	11,827–11,895	69				GCT	H
*trnL1*	11,897–11,969	73	1			TAG	H
*nad5*	11,970–13,805	1836		ATG	TAA		H
*nad6*	13,802–14,323	522	−4	ATG	TAA		L
*trnE*	14,324–14,392	69				TTC	L
*cytb*	14,395–15,535	1141	2	ATG	T		H
*trnT*	15,536–15,607	72				TGT	H
*trnP*	15,607–15,676	70	−1			TGG	L
NCR	15,703–16,607	905	26				H

Note: “L” is light strand and “H” is heavy strand (strand). Negative values indicate gene overlap (intergenic nucleotide).

## Data Availability

The mitochondria genome sequence of *A. brama orientalis* has been deposited in GenBank of NCBI (https://www.ncbi.nlm.nih.gov/, accessed on 20 March 2026), under the accession number ON123737.

## Supplementary Materials

The following supporting information can be downloaded at https://www.mdpi.com/article/10.3390/ani16071102/s1, Table S1: The best partitioning scheme selected by ModelFinder for mitochondrial genes. Table S2: Lengths of PCGs, tRNAs and rRNAs of mitogenomes in 22 Leuciscinae Species. Table S3: The RSCU of 22 Leuciscinae Species.

## Abbreviations

The following abbreviations are used in this manuscript:

CUB	Codon usage bias
PR2	Parity rule 2
RSCU	Relative synonymous codon usage
ENC	Effective number of codons
PCGs	Protein-coding genes
BI	Bayesian inference
ML	Maximum likelihood
